# Effect of *Varroa destructor*, Wounding and Varroa Homogenate on Gene Expression in Brood and Adult Honey Bees

**DOI:** 10.1371/journal.pone.0169669

**Published:** 2017-01-12

**Authors:** Gun Koleoglu, Paul H. Goodwin, Mariana Reyes-Quintana, Mollah Md. Hamiduzzaman, Ernesto Guzman-Novoa

**Affiliations:** 1 School of Environmental Sciences, University of Guelph, Guelph, Ontario, Canada; 2 Departamento de Medicina y Zootecnia en Abejas, Facultad de Medicina Veterinaria y Zootecnia, Universidad Nacional Autonoma de Mexico, Ciudad Universitaria, Mexico, Mexico; University of North Carolina at Greensboro, UNITED STATES

## Abstract

Honey bee (*Apis mellifera*) gene expression related to immunity for hymenoptaecin (*AmHym*) and defensin-1 (*AmDef-1*), longevity for vitellogenin (*AmVit2*) and stem cell proliferation for poly U binding factor 68 kDa (*AmPuf68*) was compared following *Varroa destructor* parasitism, buffer injection and injection of *V*. *destructor* compounds in its homogenate. In adults, *V*. *destructor* parasitism decreased expression of all four genes, while buffer injection decreased expression of *AmHym*, *AmPuf68* and *AmVit2*, and homogenate injection decreased expression of *AmPuf68* and *AmVit2* but increased expression of *AmDef-1* relative to their respective controls. The effect of *V*. *destructor* parasitism in adults relative to the controls was not significantly different from buffer injection for *AmHym* and *AmVit2* expression, and it was not significantly different from homogenate injection for *AmPuf68* and *AmVit2*. In brood, *V*. *destructor* parasitism, buffer injection and homogenate injection decreased *AmVit2* expression, whereas *AmHym* expression was decreased by *V*. *destructor* parasitism but increased by buffer and homogenate injection relative to the controls. The effect of varroa parasitism in brood was not significantly different from buffer or homogenate injection for *AmPuf68* and *AmVit2*. Expression levels of the four genes did not correlate with detectable viral levels in either brood or adults. The results of this study indicate that the relative effects of *V*. *destructor* parasitism on honey bee gene expression are also shared with other types of stresses. Therefore, some of the effects of *V*. *destructor* on honey bees may be mostly due to wounding and injection of foreign compounds into the hemolymph of the bee during parasitism. Although both brood and adults are naturally parasitized by *V*. *destructor*, their gene expression responded differently, probably the result of different mechanisms of host responses during development.

## Introduction

*Varroa destructor* is the most deleterious parasitic mite of the honey bee, *Apis mellifera* [1]. For example, *V*. *destructor* infestations were associated with 85% of colony losses examined over the winter of 2009 in Ontario, Canada [2]. Bee brood parasitized by *V*. *destructor* developed into adults with shorter abdomens, deformed wings and shorter life-spans [3, 4], and parasitized foragers were more likely to get lost and drift between colonies [5]. Behavioural mechanisms of resistance against *V*. *destructor* and genes associated with them have been identified in some honey bee genotypes [6–8], but the majority of honey bee genotypes express these behaviours at low levels, and thus most colonies are highly susceptible to parasitism by *V*. *destructor*.

One negative impact of *V*. *destructor* is the direct physical damage of the honey bee during feeding. *V*. *destructor* females puncture the cuticle of immature or adult honey bees with their chelicerae, creating an open wound, injecting saliva into the haemolymph and then using their hypostome to feed on the honey bee’s haemolymph [9]. Another effect is the vectoring/activation of honey bee viruses, such as deformed wing virus (DWV) and Kashmir bee virus (KBV), which can be transmitted in the saliva of *V*. *destructor* through its chelicerae [10, 11]. In addition, *V*. *destructor* parasitism may suppress expression of numerous honey bee genes. For example, *V*. *destructor* suppressed expression of genes in the immune system, like those for the antimicrobial peptides (AMPs), defensin-1 and hymenoptaecin, as well as genes related to longevity and development, like the storage protein, vitellogenin, and the putative cell proliferation regulator, poly U binding factor 68 kDa (also known as Half Pint or pUf68) [12, 13].

One factor that can affect honey bee gene expression is its developmental stage. For example, vitellogenin expression first reaches detectable levels late in brood development and then expression declines progressively with age in adults [14]. Developmental stage can also affect responses to stress. For example, adults have more peptidoglycan recognition protein-S2, carboxylesterase and phenol oxidase following septic and aseptic injury, whereas injury does not change the levels in brood [15]. The authors speculated that adults use a wider variety of immune related molecules to defend themselves compared to brood.

The objective of this study was to compare the effects of *V*. *destructor* parasitism to the effects produced by other stresses that share certain aspects of parasitism by *V*. *destructor* through an examination of the expression of several genes in brood and adult honey bees. Injecting a buffer solution would create injury, which could have impacts like the injury caused by *V*. *destructor*‘s chelicerae, and injecting a buffer solution containing a soluble homogenate of *V*. *destructor* could create additional stress by also introducing foreign compounds into the honey bee’s haemolymph like the foreign compounds injected in the varroa mite’s saliva. The genes chosen were defensin-1 (*AmDef-1*), hymenoptaecin (*AmHym*), poly U binding factor 68 kDa (*AmPuf68*) and vitellogenin (*AmVit2*) as each has been shown to be down-regulated by *V*. *destructor* parasitism of adults [13, 16–18]. In addition, *AmDef-1*, *AmHym*, *AmPuf68* and *AmVit2* were chosen because they are linked to different aspects of honey bee stress responses, specifically the Toll immune pathway, Imd immune pathway, epithelium wound response and overall honey bee health, respectively [19–21]. With this experimental design, the relationship of *V*. *destructor* parasitism and several aspects of its parasitism could be examined and compared between developmental stages.

## Materials and Methods

### Ethics statement

No permits were required to conduct the research or analyses. The research and analyses were conducted under the supervision of researchers of the Honey Bee Research Center, University of Guelph in Guelph, ON, Canada. Beekeeping and breeding were performed in accordance with the University and Ontario Ministry of Agriculture, Food and Rural Affairs (OMAFRA) bio-safety regulations.

### Source of honey bees, *V*. *destructor* and homogenate

European honey bees of the Buckfast strain were reared at the Honey Bee Research Center of the University of Guelph in Guelph, ON, Canada. Queen bees of this genotype were bred in isolation on Thorah Island, ON, Canada to ensure the purity of the strain. Eight honey bee source colonies were selected and treated with fluvalinate strips (Apistan®, Novartis, Mississauga, ON, CA) for six weeks prior to the experiments to control *V*. *destructor* infestations.

Adult varroa mites from heavily infested colonies that had not been treated with miticides for at least six months, were harvested from brood cells using a fine paint brush and were temporarily placed into Petri dishes (Fisher Scientific, Fair Lawn, NJ, USA). Varroa mites required for preparation of *V*. *destructor* homogenate were processed immediately as described below, whereas those that were used for artificial infestation of bees were starved for 3–4 h prior to use.

To prepare homogenate, collected varroa mites were washed with PBS (0.038 M anhydrous monosodium phosphate, 0.162 M disodium phosphate, 0.75 M sodium chloride, pH 7.4) by vortexing for 15 s. After washing, the PBS was removed, and approximately 100 varroa mites were blotted dry and placed in a sterile mortar with 5 μL of PBS per mite. *Varroa destructor* were ground until no visible particles of their exoskeleton remained. The resulting homogenate was centrifuged at 16,500 g for 10 min, and the supernatant removed and stored at -20°C.

### Treatments

To obtain adult bees, frames containing emerging brood were taken from honey bee source colonies and incubated overnight inside screened emergence cages (5 x 28 x 25.5 cm) at 32–35°C, and 60% RH. Sixty-four to 70 newly emerged adult bees were used for each treatment. For buffer injection, adults were injected with 2.5 μL of PBS, which has previously been used as a control for injections of honey bees [15, 22]. Injections were performed using a 32 gauge syringe needle between the second and third tergite. For *V*. *destructor* homogenate injection, adults were injected with 2.5 μL of *V*. *destructor* homogenate in PBS. Adults of each treatment were placed in a screened hoarding cage (12.7x8.5x14.5 cm; with 3 mesh/cm, wire screened wall) and fed 50% sucrose solution and water *ad libitum* and incubated at 32–35°C and 60% RH for 48 h. As a control, non-injected and non-treated adult bees were handled and incubated similarly. For *V*. *destructor* infestation, adults were placed in Benton queen cages (8 honey bees per cage). Two varroa mites per bee were placed on each adult through the cage screen using a fine brush. Control adults were incubated similarly in Benton queen cages but without the addition of varroa mites. The adults were fed queen candy *ad libitum* and watered by spreading drops of water on the queen cage screen twice a day. The cages were incubated at 32–35°C and 60% RH for 2 days. For adults, larger hoarding cages were selected as the control of the buffer and homogenate injections, while smaller Benton queen cages were used as the control for parasitism. The larger hoarding cages would presumably provide more space for the honey bees following treatment, thus reducing the amount of stress, while the smaller queen cages would make it easier for varroa mites to locate their host and thus increase the chances of parasitism.

Combs containing white-eyed pupae (hereafter referred to as brood) from source colonies were used for brood experiments. The cells of 50 brood for each treatment were opened with a razor folding back the wax capping, and then the exposed brood were treated as follows. For buffer injection, brood were pierced with a 32 gauge Hamilton syringe (Fisher Scientific, Fair Lawn, NJ, USA) on the dorsal side of the thorax, approximately 2 mm behind where the thorax meets the head and then 2.5 μL of PBS was injected. For *V*. *destructor* homogenate injection, brood were treated as per the buffer injection, except that they were injected with *V*. *destructor* homogenate. For *V*. *destructor* infestation, brood were artificially infested by placing one live *V*. *destructor* onto each brood body using a fine paint brush. After treatment, the cappings were closed and sealed with molten beeswax using a fine paint brush. The cappings were then marked using water based, non-toxic paint (L551P2, Hunt Int., Mississauga, ON, CA) for identification. The time to handle brood cells from uncapping to recapping was approximately 2 min. The non-injected control for these treatments were capped brood cells that were opened and closed for about the same amount of time as that of the injected treatments, verifying at the same time, the absence of varroa mites in the cells before re-sealing them [18]. Following the treatments, combs with treated brood were placed in emergence cages and incubated at 32–35°C and 60% RH for 48 h. Three replicates of all the above experiments were conducted.

### Sample collection, RNA extraction and cDNA synthesis

For controls and treatments, six adult or brood samples were collected at 0, 2, 12, 24 and 48 h post treatment (hpt) and immediately frozen at -70°C. Dead adult bees or mortally injured brood were not sampled and were excluded from the study. Brood was deemed mortally injured if they lost their shape and rigidity and/or bled haemolymph significantly. Less than 5% bee mortality was observed during the experimental period of 48 h. Additionally, only adults or brood with *V*. *destructor* that were still attached at the sampling times were collected to ensure that the *V*. *destructor* infestation resulted in parasitism. Also, varroa mites were removed from the adult or brood to observe that they were alive by observing if they moved their legs when flipped upside down and probed with a paintbrush to test that *V*. *destructor* parasitism had occurred.

Total RNA was extracted from three adults or brood pooled for each time point per treatment as per Chen et al. [23]. Samples were macerated with a mortar and pestle in liquid nitrogen, transferred into a 2 mL microcentrifuge tube and then 900 μL of extraction buffer (0.2 M Tris, 0.4 M potassium chloride, 0.2 M sucrose, 0.035 M magnesium chloride hexahydrate, 0.025 M EDTA, pH 9.0) was added. Then, 900 μL of phenol:chloroform:isoamyl alcohol (25:24:1) were added to each tube, the tubes were vortexed for 1 min, and centrifuged at 28,000 g for 15 min. After centrifugation, the supernatant from each tube was transferred and mixed with an equal volume of chloroform:isoamyl alcohol (24:1). Following mixing and centrifugation at 28,000 g for 5 min, the supernatant was transferred and mixed with one-fourth its volume of LiCl and stored overnight at 4°C. The samples were then centrifuged at 28,000 g for 5 min, and the RNA pellet was rinsed with 70% ethanol, dried, and re-suspended in 25 μL of autoclaved dH_2_O. RNA samples were stored at -70°C.

cDNA was prepared using a RevertAid™ H Minus First Strand cDNA Synthesis Kit (Fermentas, Burlington, ON, CA) following the manufacturer’s instructions. The cDNA was stored at -20°C.

### Primers

Primer sequences for the constitutively expressed housekeeping genes, *RpS5* and *GAPD2*, were obtained from Thompson et al. [24]. The target genes, *AmHym and AmDef-1*, were obtained from Evans [25], whereas primer sequences for *AmPuf68* were obtained from Hamiduzzaman et al. [18] and *AmVit2* from Guidugli et al. [26]. Additionally, the relative amounts of deformed wing virus (DWV), black queen cell virus (BQCV), Kashmir bee virus (KBV), sac brood virus (SBV), acute bee paralysis virus (ABPV) and Israeli acute paralysis virus (IAPV) were screened. Primer sequences related to analyze honey bee gene expression and viruses are listed in Table 1. The primers were ordered from Laboratory Services of the University of Guelph (Guelph, ON, CA).

**Table 1:** Primers used for amplification of the target and constitutive control genes, and honey bee viruses.

Target	Designation	Forward (F) and reverse (R) primers (5’–3’)	Product length	Source
Defensin-1	AmDef-1*	F: TGCGCTGCTAACTGTCTCAG	119 bp	Evans [25]
		R: AATGGCACTTAACCGAAACG		
Hymenoptaecin	AmHym*	F: CTCTTCTGTGCCGTTGCATA	200 bp	Evans [25]
		R: GCGTCTCCTGTCATTCCATT		
Poly-U-binding factor 68 kDa	AmPuf68*	F: CAAGACCTCCAACTAGCATG	201 bp	Hamiduzzaman et al. [18]
		R: CAACAGGTGGTGGTGGTG		
Vitellogenin	AmVit2*	F: ACGACTCGACCAACGACTT	494 bp	Guidugli et al. [26]
		R: ACGAAAGGAACGGTCAATTCC		
Ribosomal Protein S5	RpS5**	F: AATTATTTGGTCGCTGGAATTG	115 bp	Thompson et al. [24]
		R: TAACGTCCAGCAGAATGTGGTA		
Glyceraldehyde 3-phosphate dehydrogenase2	GAPD2**	F: GATGCACCCATGTTTGTTTG	203 bp	Thompson et al. [24]
		R: TTTGCAGAAGGTGCATCAAC		
Deformed Wing Virus	DWV***	F: ATCAGCGCTTAGTGGAGGAA	642 bp	Hamiduzzaman et al. [28]
		R: ATAGATATCAGTCAACGGAGC		
Black Queen Cell Virus	BQCV***	F: GTCAGCTCCCACTACCTTAAAC	698 bp	Hamiduzzaman et al. [28]
		R: AACAAGAAGAAACGTAAACCAC		
Israeli Acute Paralysis Virus	IAPV***	F: AGACACCAATCACGGACCTCAC	138 bp	Maori et al. [40]
		R: GAGATTGTTTGAGAGGGGTGG		
Sac Brood Virus	SBV***	F: GGATGAAAGGAAATTACCAG	426 bp	Tentcheva et al. [41]
		R: CCACTAGGTGATCCACACT		
Kashmir Bee Virus	KBV***	F: GATGAACGTCGACCTATTGA	414 bp	Stoltz et al. [42]
		R: TGTGGGTTGGCTATGAGTCA		
Acute Bee paralysis Virus	ABPV***	F: CCCAACGCACAAACAAATA	516 bp	Fedorova et al. [43]
		R: CTCCAGACAACAACAACAA		

* Honey bee gene

** Constitutive honey bee control

*** Honey bee virus.

### Semi-quantitative relative RT-PCR

PCR reactions were run in an Eppendorf AG 22331 Master Cycler (Eppendorf, Hamburg, DE). Each 15 μl reaction contained 2 μl of cDNA, 5 units (1 μl) of *Taq* DNA polymerase (New England Biolabs, Pickering, ON, CA), 10X *Taq* reaction buffer, 1 μl 10 mM dNTPs and 1 μM of each primer for both the honey bee target gene and either the constitutive housekeeping gene *RpS5* or *GAPD2*. One reaction was performed with RNA directly without cDNA synthesis, as a negative control. Reaction conditions were 35 cycles of 30 s at 94°C, 60 s at 58°C and 60 s at 72°C, and a final extension step at 72°C for 10 min. PCR products were separated on a 1% TAE agarose gel with 1% ethidium bromide and visualized using a BioDoc-It ™ Imaging System (UVP, Mississauga, ON, CA) under UV light. The intensity of the amplified bands was quantified in pixels using the Scion Image (Scion Corporation, Frederick, MD, USA) as per Dean et al. [27]. Relative expression units (REUs) were determined from the ratio of intensity of the band of the target gene to that of the band of the constitutive control gene (honey bee housekeeping gene) as per Hamiduzzaman et al. [18]. The intensity of the bands of the constitutive gene was consistent at all time points. To determine whether quantification at 35 amplification cycles was not affected by signal saturation of the band intensities, randomly selected samples with high, medium and low REUs were also quantified in the same manner with five and ten fewer amplification cycles, and the pattern of expression based on the REU values were not significantly different when 25, 30 and 35 amplification cycles were used (F_2,15_ = 0.30, p = 0.75). An example of one of the gel pictures is shown of co-amplification of the PCR products for *AmHym* and *RpS5* used to estimate relative expression in brood and adult bees in response to buffer injection (S1 Fig).

The same RNA was used for honey bee gene expression was also used to determine the levels of DWV, BQCV, IAPV, SBV, ABPV and KBV following the same treatments [28]. The amounts of each honey bee virus relative to a bee constitutive control gene (ribosomal protein RpS5) were determined by a multiplex reverse transcription-PCR (RT-PCR). Multiplex simultaneous reactions were done for each virus combining one set of virus-specific primers with the RpS5 primers [28]. All PCR reactions were done with a Mastercycler (Eppendorf, Mississauga, ON, CA). Each 15 μl of reaction contained 1.5 μl of 10x PCR buffer (New England BioLabs), 0.5 μl 10 mM of dNTPs (Bio Basic Inc., Markham, ON, CA), 1 μl of 10 μM forward and reverse primers for the RpS5 gene and 10 μM forward and reverse primers for one of the honey bee viruses, 0.2 μl 5U/μl of *Taq* polymerase (New England BioLabs), 1 μl of the cDNA sample, and 7.8 μl of dd H_2_O. For IAPV, SBV, ABPV and KBV, the PCR conditions were 94°C for 3 min, followed by 35 cycles of 30 s at 94°C, 60 s at 55°C and 60 s at 72°C, and a final extension step at 72°C for 10 min. Amplification conditions for DWV and BQCV were the same, except that the annealing temperature was 58°C. In case of RT-negative control, there was no DNA to be amplified, and thus the reaction did not produce any bands.

### Statistical analyses

For each time point, the mean and standard errors were calculated from three biological and two technical replications. To test for differences in relative expression units (REU) of the target genes (*AmDef-1*, *AmHym*, *AmPuf68* and *AmVit2*) between time points within a treatment, analysis of variance (ANOVA) was performed. Additionally, a correlation was made between the relative levels of infection levels of the detectable honey bee viruses (DWV, BQCV, IAPV and SBV) of the same samples [28] and the REU of honey bee target genes in this study. Viruses that were undetectable in the samples (ABPV and KBV) were excluded from the analysis. The ANOVA and linear regressions were performed with the package IBM-SPSS v. 23 (SPSS Inc., Chicago, IL, USA). Analysis of covariance (ANCOVA) was used to assess the homogeneity of regression slopes for changes in REU over time to compare gene expression patterns between treatments over the course of the experiment. ANCOVA was performed using the program, XLSTAT Version 2016.02.27390 (Addinsoft, New York, NY, USA).

## Results

### *AmDef-1* expression

**Fig 1:**
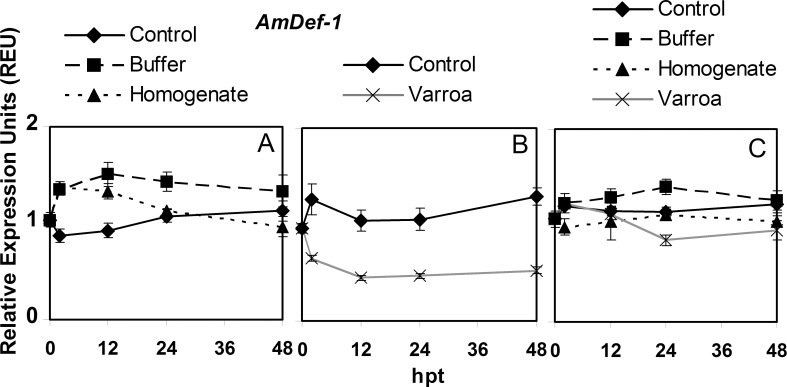
Semi-quantitative expression of *AmDef-1* relative to *GAPD2* in bees in response to *Varroa destructor* parasitism, buffer injection and injection of *V*. *destructor* homogenate from 0 to 48 hours post treatment (hpt). The panels are: A) adult bees in hoarding cage, B) adult bees in Benton queen cage, and C) brood in comb. The values of relative expression units presented are means ± SE.

For control adult bees in hoarding cages, *AmDef-1* expression was not significantly different at any time point over the course of the experiment, which was also true for the buffer injection treatment (Fig 1A, S1 Table). For *V*. *destructor* homogenate injection, there was a significant increase in expression at 2 hpt, which then decreased and returned to levels so that 0 and 48 hpt values were not significantly different. For control adults in queen cages, there were no significant differences over time in *AmDef-1* expression (Fig 1B, S1 Table). The expression of *AmDef-1* significantly decreased by *V*. *destructor* parasitism at 2 and 12 hpt, but then remained relatively unchanged for the remainder of the experiment. In brood, *AmDef-1* expression for control, buffer or homogenate injections did not significantly change over time. Although expression with parasitism of brood by *V*. *destructor* fluctuated, it was never significantly different from 0 hpt. Only expression at 2 hpt was significantly higher than at 24 and 48 hpt (Fig 1C, S1 Table).

The pattern of *AmDef-1* expression over the course of the experiment in control adult bees was not significantly different between the hoarding and queen cages (S2 Table). The lowest overall expression was with *V*. *destructor* parasitism, which was significantly different compared to buffer and homogenate injection. Homogenate injection also resulted in a pattern of significantly lower *AmDef-1* expression than buffer injection. In brood, the overall pattern of *AmDef-1* expression with *V*. *destructor* parasitism was also the lowest among the treatments. It was not significantly different from the pattern with homogenate injection but was for buffer injection. Both buffer and homogenate injection were not significantly different from the control.

### *AmHym* expression

**Fig 2:**
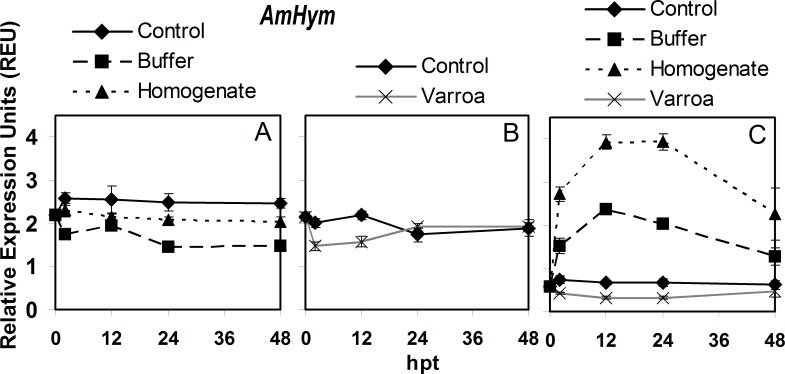
Semi-quantitative expression of *AmHym* relative to *RpS5* in bees in response to *Varroa destructor* parasitism, buffer injection and injection of *V*. *destructor* homogenate from 0 to 48 hours post treatment (hpt). The panels are: A) adult bees in hoarding cage, B) adult bees in Benton queen cage, and C) brood in comb. The values of relative expression units presented are means ± SE.

For control adult bees in hoarding cages, *AmHym* expression did not significantly differ at any time during the experiment (Fig 2A, S1 Table). However, buffer injection resulted in significant decreases in expression at 2 and then again at 24 hpt. For *V*. *destructor* homogenate injection, expression did not change significantly at any of the time points in the experiment. For control adults in queen cages, *AmHym* expression also did not change significantly over time (Fig 2B, S1 Table). However, *V*. *destructor* parasitism caused *AmHym* expression to decrease significantly at 2 and 12 hpt, then returning to similar levels to those of 0 hpt.

For brood, *AmHym* expression showed no significant changes over time (Fig 2C, S1 Table). In contrast, buffer injection resulted in a rapid and significant increase in expression at 2 hpt, peaking at 12 and 24 hpt, but then returning to the same 0 hpt level by 48 hpt. Homogenate injection had a similar significant effect on expression at the same time points but to a greater degree. Parasitism by *V*. *destructor* resulted in significantly lower levels of *AmHym* expression at 12 and 24 hpt, but it increased by 48 hpt and was not significantly different from 0 hpt.

For *AmHym*, the overall expression pattern in control adult bees was significantly different between hoarding and queen cages making it important to compare the expression patterns to their respective cage controls (S2 Table). The *AmHym* expression pattern over the course of the experiment was significantly lower in bees with *V*. *destructor* parasitism and buffer injection compared to their respective controls. The pattern of expression with homogenate injection in adults was significantly higher than the buffer treatment but lower than its control. In brood, the expression pattern of *AmHym* was not significantly affected by *V*. *destructor* parasitism compared to the control, but both of those were different from the expression pattern with buffer and homogenate injection, which were also different from each other.

### *AmPuf68* expression

**Fig 3:**
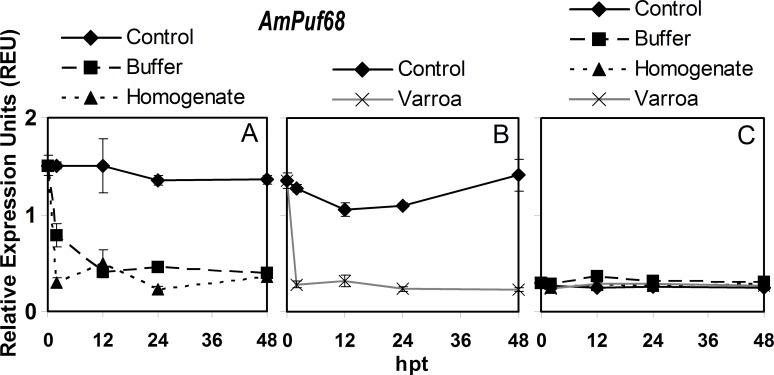
Semi-quantitative expression of *AmPuf68* relative to *RpS5* in bees in response to *Varroa destructor* parasitism, buffer injection and injection of *V*. *destructor* homogenate from 0 to 48 hours post treatment (hpt). The panels are: A) adult bees in hoarding cage, B) adult bees in Benton queen cage, and C) brood in comb. The values of relative expression units presented are means ± SE.

A comparison between different time points showed that for control adult bees in hoarding cages, *AmPuf68* expression did not change significantly over time (Fig 3A, S1 Table). Buffer injection resulted in a significant sharp decrease in expression at 2 hpt, and then expression did not significantly change at any time point for the rest of the experiment. Similar results were observed for *V*. *destructor* homogenate injection. For control adults in the queen cages, the only significant difference in *AmPuf68* expression was at 24 hpt which was lower than that at 2 hpt (Fig 3B, S1 Table). Parasitism by *V*. *destructor* produced a large significant decrease of 79.3% in expression from 0 to 2 hpt and then expression did not significantly change over time. In brood, *AmPuf68* expression was not significantly different over time for the control or any of the treatments (Fig 3C, S1 Table).

For *AmPuf68*, the expression patterns for the treatments in adult bees over the course of the experiment should be compared to their respective cage controls as the patterns were significantly different between the controls with the hoarding versus queen cages (S2 Table). The overall pattern of *AmPuf68* expression during the experiment was significantly lower in adult bees with buffer, homogenate and parasitism compared to their respective controls. Parasitism of adult bees by *V*. *destructor* had the most negative effect on expression compared to its control, and the lowering in expression relative to their respective controls was similar between homogenate injection and *V*. *destructor* parasitism. However, the pattern of *AmPuf68* expression over the experiment with homogenate injection in adult bees was lower than the pattern with buffer injection. In brood, the only significantly difference in the overall pattern of *AmPuf68* expression was between buffer injection and the control (S2 Table).

### *AmVit2* expression

**Fig 4:**
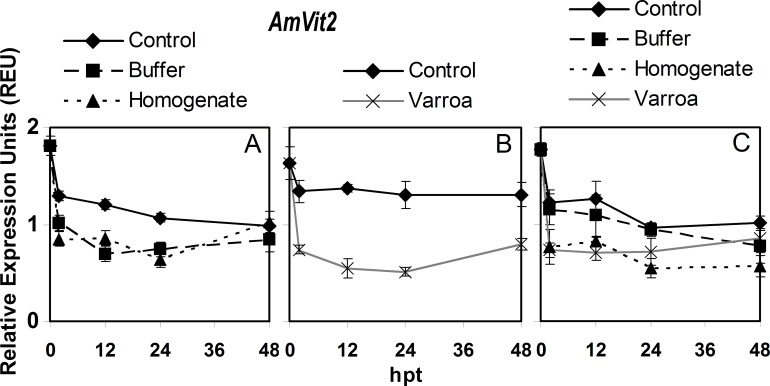
Semi-quantitative expression of *AmVit2* relative to *RpS5* in bees in response to *Varroa destructor* parasitism, buffer injection and injection of *V*. *destructor* homogenate from 0 to 48 hours post treatment (hpt). The panels are: A) adult bees in hoarding cage, B) adult bees in Benton queen cage, and C) brood in comb. The values of relative expression units presented are means ± SE.

For the hoarding cage control, expression of *AmVit2* in adult bees decreased significantly at 2 hpt and continued to slowly decrease significantly until 48 hpt (Fig 4A, S1 Table). Buffer injection decreased *AmVit2* expression significantly at 2 and 12 hpt and then expression remained low. Homogenate injection produced very similar changes in expression as buffer injection, except for a significant increase from 24 to 48 hpt. For the queen cage control adult bees, there were no significant differences over time (Fig 4B, S1 Table). However, *V*. *destructor* parasitism significantly decreased *AmVit2* expression by 54.6% between 0 and 2 hpt, then remained relatively unchanged for the rest of the experiment. In brood, the expression of *AmVit2* decreased over time with expression levels at 24 and 48 hpt being significantly lower than those at 0 hpt (Fig 4C, S1 Table). Buffer injection resulted in brood having very similar changes in *AmVit2* expression as that of the control. However, homogenate injection as well as *V*. *destructor* parasitism both resulted in a similar significant sharp drop in *AmVit2* expression at 2 hpt, and then expression remained relatively unchanged over time.

For *AmVit2*, the expression pattern in control adult bees over the course of the experiment was not significantly different between hoarding and queen cages (S2 Table). The overall expression patterns of *AmVit2* for *V*. *destructor* parasitism, buffer injection and homogenate injection was significantly lower than their respective controls, but not significantly different from each other. In brood, the overall expression pattern of *AmVit2* was not significantly different between buffer injection and the control, and also not significantly different between homogenate injection and *V*. *destructor* parasitism.

### Relationship of honey bee gene expression to viral levels

The brood and adult samples used for measuring gene expression in this study were also used to determine the levels of six honey bee viruses, DWV, BQCV, IAPV, SBV, ABPV and KBV. None of those viruses, except IAPV, were detected in control bees, while ABPV and KBV were never detectable. However, levels of DWV and BQCV increased over time in both adult bees and brood after *V*. *destructor* parasitism or homogenate injection, whereas SBV was detected only in homogenate injected bees. Correlation analyses between the levels of the viruses detected following *V*. *destructor* parasitism and homogenate injection treatments and the level of gene expression showed no significant relationship between relative expression levels of *AmDef-1*, *AmHym*, *AmPuf68* or *AmVit2* with the levels of the detectable viruses (DWV, BQCV, IAPV and SBV) in adults and brood (S2–S5 Figs).

## Discussion

As previously reported [13, 16–18], this study also showed that *V*. *destructor* parasitism resulted in decreased expression of *AmDef-1*, *AmHym*, *AmPuf68* and *AmVit2* relative to its cage control at one or more time points for both adults and brood, except for *AmPuf68* and *AmDef-1* in brood. Previous studies that have found suppression of five different defense genes by *V*. *destructor* parasitism in adults [12] and two defense genes by *V*. *destructor* parasitism in brood [16] support that the parasite causes immunosuppression of bees. The idea of immunosuppression during varroa parasitism is also supported by Kanbar and Engels [9], who showed that *V*. *destructor* can maintain open wounds in the honey bee integument and repeatedly feed on the same open wounds over a long period of time, and by Richards et al. [29], who showed that saliva of *V*. *destructor* can physically damage haemocytes. However, immunosuppression by *V*. *destructor* was not supported by another study showing that expression of nine defense genes was increased by wounding but none were affected by *V*. *destructor* parasitism in brood [30].

This study showed that wounding followed by buffer injection or varroa mite homogenate injection also affected bee gene expression with different impacts depending upon which bee gene was examined. For example, buffer injection of adults resulted in significant decrease in expression of *AmPuf68* and *AmVit2* at 2 hpt relative to their cage controls that remained suppressed during the experiment, similar to homogenate injection and *V*. *destructor* parasitism relative to their respective cage controls. In the case of *AmDef-1* expression, however, the effect of buffer injection was quite different from that of homogenate injection and *V*. *destructor* parasitism relative to their respective cage controls. This shows the challenge in making conclusions about immunosuppression and the value of examining multiple genes under a variety of treatments like buffer injection, homogenate injection and *V*. *destructor* parasitism in order to determine if the effects of parasitism are related to wounding or are distinct from it.

For *V*. *destructor* parasitism of adults, *AmPuf68* and *AmVit2* expression were the most strongly suppressed among the four genes tested compared to their cage controls. The amount of decreased *AmPuf68* and *AmVit2* expression with *V*. *destructor* parasitism was consistent with that reported by Dainat et al. [17] and Hamiduzzaman et al. [18]. In contrast, parasitism by *V*. *destructor* caused much less of a reduction in *AmDef-1* and *AmHym* expression in adults relative to their cage controls. Vitellogenin is the main storage protein of honey bees and is involved in hormonal regulatory pathways related to longevity and overall honey bee health [17, 19, 31], whereas *AmPuf68* encodes a 68kDa poly U RNA-binding protein that is related to a *Drosophila melanogaster* protein controlling stem cell proliferation for the renewal of the epithelium following tissue wounding [21, 32, 33]. However, both *AmDef-1* and *AmHym* are AMPs that permeabilize bacterial membranes, but *AmDef-1* is mainly regulated by the Toll pathway that is activated by a variety of fungal and bacterial pathogen associated molecular patterns (PAMPs), while *AmHym* is mainly regulated by the Imd pathway that is activated by the PAMP, peptidoglycan [20, 34, 35, 36]. Therefore, parasitism of adult bees by *V*. *destructor* appeared to have affected the overall health (*AmVit2* expression) and regulation of epithelium renewal (*AmPuf68* expression) relatively more than it affected the immune AMP responses (*AmDef-1* and *AmHym* expression) over the time period used in this study. A relatively strong effect on overall bee health and a bee wound response could be expected from a parasite that is as damaging as *V*. *destructor* that punctures the body of the bee to feed.

Expression patterns of the two AMP genes in this study, *AmDef1* and *AmHym*, were significantly different between buffer injection and homogenate injection in adults. Both of those treatments would cause wounding, but changes in expression with homogenate injection reflects both the impact of piercing the bee integument and introducing foreign compounds into the honey bee haemolymph. Compared to the cage control, expression of *AmDef-1* was lower with homogenate than buffer injection, whereas the reverse was true for *AmHym* expression. This suggests that there are bioactive elements in the homogenate that can affect the expression of at least those two AMP genes.

One limitation in the study about adults was that two cage types were used. Although the cage type had no effect on the expression patterns of *AmDef1* and *AmVit2*, it did have different impacts on *AmHym* and *AmPuf68* expression patterns over the course of the experiment. Therefore, it is important to view the expression patterns with different treatments relative to their respective cage controls. Despite these limitation about comparisons between some treatments for their effects on *AmHym* and *AmPuf68* expression patterns, it was notable over the course of the experiment how relatively similar the patterns were between buffer injection, homogenate injection and *V*. *destructor* parasitism for *AmPuf68* and *AmVit2* expression, compared to how relatively different the patterns were for *AmHym* and *AmDef1* expression.

For brood, the control involved only opening and closing brood cells and had no significant effects on gene expression, except for a rapid decrease in *AmVit2* expression like in control adults. Expression of *AmVit2* thus appears to be highly sensitive to any handling of the bees, whether caging or opening and recapping cells. Even for that gene, however, there were statistically significant differences produced by the treatments.

Buffer injection of brood increased expression of *AmHym* and *AmPuf68* compared to the control. In contrast, buffer injection did not have an effect on *AmDef-1* and *AmVit2* expression, unlike in adults. Thus, brood appears to respond quite differently than adults to wounding. Expression of *AmHym* was also significantly elevated by injection of saline in brood [12], and wounding of brood was also reported to increase *AmDef-2* and *AmHym* expression by Kuster et al. [30]. Injection of *V*. *destructor* homogenate into brood resulted in significantly higher *AmHym* and significantly lower *AmVit2* expression compared to the control. This response of *AmHym* was similar to the increased *AmHym* expression following the injection of heat-killed *E*. *coli* into brood, although the increased expression in that case was not greater than injecting saline [12]. Injection of *V*. *destructor* homogenate into brood was significantly different from buffer injection in brood for *AmDef-1*, *AmHym* and *AmVit2*, indicating that a number of genes respond to bioactive compounds in the homogenate that were injected in buffer.

*Varroa destructor* parasitism of brood resulted in significantly lowered expression of *AmDef-1* and *AmVit2* relative to the control. This was a similar, but in general a lesser effect than that observed in adults. No changes were observed in the brood for *AmHym* and *AmPuf68* expression with parasitism, unlike the decreases in expression observed in adults, indicating that those genes in brood do not respond like in adults. However, it is important to note that the basal level of *AmPuf68* expression (at 0 hpt) was much lower in brood than in adults, and so that gene may play a less important role in brood than in adults and less useful for comparison. The effect of *V*. *destructor* parasitism of brood on *AmVit2* expression was not significantly different from buffer injection, and therefore could mostly be due to wounding by *V*. *destructor*. Also, the effect of *V*. *destructor* parasitism of brood on *AmDef-1* expression was not significantly different from homogenate injection, and thus could mostly be due to the introduction of foreign compounds into the haemolymph of the bee.

It could be argued that the changes in gene expression in this study were being affected by viruses that were inoculated into the bees following injection of varroa homogenate or by varroa parasitism. However, there was no relationship between virus levels and the changes in expression of any of the genes in this study. In a previous study using the same RNA from the same collection of bees it was shown that among six viruses screened, DWV, BQCV, SBV and IAPV were not detected in buffer control bees, except for low levels of IAPV [28]. However, the levels of those viruses increased in both adult bees and brood after homogenate injection or *V*. *destructor* parasitism. While strand-specific RT-PCR was not done in that study to determine if the replicative strands of the different viruses were detectable to confirm viral replication, the large increases in virus levels over time by the injection of varroa homogenate and varroa parasitism indicates the varroa mites contained the viruses. Previous reports of the effect of bee viruses on bee gene expression have been variable. For example, expression of several AMP genes in honey bees was up-regulated following DWV infection of brood [37], while DWV infection, vectored by *V*. *destructor*, adversely affected humoral and cellular immune responses in bees [38]. However, Azzami et al. [39] reported that the levels of AMPs were not elevated following artificial inoculation with ABPV. Part of the differences between studies could be due to differences in honey bee genotypes, strains of the virus, methods of introducing the virus as well as other factors. At least from this study, it appears that the changes in gene expression observed were not correlated with any of the viruses detected in the honey bees regardless of whether the viruses were introduced naturally by *V*. *destructor* parasitism or artificially by homogenate injection.

This study indicates that the effects of *V*. *destructor* parasitism on bee gene expression may in some cases reflect wounding or reflect the introduction of foreign compounds into the bee haemolymph. Only the effect of *V*. *destructor* parasitism on *AmDef-1* expression in adults could be considered to be consistent with immunosuppression by the parasite. However, an examination of more genes is needed to show how representative the results are from this work. Also, this study used a homogenate composed of all the buffer soluble compounds from a varroa mite and likely contains a number of bio-active compounds. Thus, further work is needed to purify, assay and identify these compounds. The results with *AmHym* expression in brood indicates that it may be a good gene to assay for bio-activity during effector purification as its induction was easily distinguishable from that of injecting buffer. Studying purified bio-active *V*. *destructor* compounds would help reveal their function and the likelihood that they could enter the honey bee haemolymph during parasitism. Eventually, counteracting or inactivating such compounds may provide a new, highly effective and specific way to control *V*. *destructor* parasitism.

## Supporting Information

S1 Fig Gel picture of the co-amplification of *AmHym* and the housekeeping gene, *RpS5*, used to estimate relative expression in brood (A) and adult bees (B) in response to buffer injection at different hours post treatment (hpt). Lanes 1–5 show the control treatment at 0, 2, 12, 24 and 48 hpt, respectively. Lane 6 shows negative control with no DNA. Lane M (far left) is a 100 bp DNA ladder for both panels.
(TIF)

S2 Fig Correlation between relative expression units of *AmDef-1*, *AmHym*, *AmPuf68*, *AmVit2* and the relative quantification units of deformed wing virus (DWV) (relative to a constitutive housekeeping gene) in adult bees and brood in response to *Varroa destructor* parasitism, and injection of *V*. *destructor* homogenate from 2 to 48 hours post treatment (hpt). The panels are relative expression units of *AmDef-1*, *AmHym*, *AmPuf68*, *AmVit2* in adult bees (A, B, C, D) and in brood (E, F, G, H), respectively. Thirty-two samples with different levels of viral quantification were used to generate the linear regression line and equation.
(TIF)

S3 Fig Correlation between relative expression units of *AmDef-1*, *AmHym*, *AmPuf68*, *AmVit2* and the relative quantification units of black queen cell virus (BQCV) (relative to a constitutive housekeeping gene) in adult bees and brood in response to *Varroa destructor* parasitism, and injection of *V*. *destructor* homogenate from 2 to 48 hours post treatment (hpt). The panels are relative expression units of *AmDef-1*, *AmHym*, *AmPuf68*, *AmVit2* in adult bees (A, B, C, D) and in brood (E, F, G, H), respectively. Twenty-four samples with different levels of viral quantification were used to generate the linear regression line and equation.
(TIF)

S4 Fig Correlation between relative expression units of *AmDef-1*, *AmHym*, *AmPuf68*, *AmVit2* and the relative quantification units of Israeli acute paralysis virus (IAPV) (relative to a constitutive housekeeping gene) in adult bees and brood in response to *Varroa destructor* parasitism, and injection of *V*. *destructor* homogenate from 2 to 48 hours post treatment (hpt). The panels are relative expression units of *AmDef-1*, *AmHym*, *AmPuf68*, *AmVit2* in adult bees (A, B, C, D) and in brood (E, F, G, H), respectively. Twenty-four samples with different levels of viral quantification were used to generate the linear regression line and equation.
(TIF)

S5 Fig Correlation between relative expression units of *AmDef-1*, *AmHym*, *AmPuf68*, and *AmVit2* and the relative quantification units of sac brood virus (SBV) in adult bees and brood in response to *Varroa destructor* parasitism, and injection of *V*. *destructor* homogenate from 2 to 48 hours post treatment (hpt). The panels are relative expression units of *AmDef-1*, *AmHym*, *AmPuf68*, *AmVit2* in adult bees (A, B, C, D) and in brood (E, F, G, H), respectively. Twenty-four samples with different levels of viral quantification were used to generate the linear regression line and equation.
(TIF)

S1 Table Analysis of variance (ANOVA) of relative expression units (REU) on gene expression in European bees at different time points. Buffer and homogenate injection treatments of adult bees were performed in hoarding cages with a non-treated control. *Varroa* parasitism of adult bees was studied in queen cages with a non-treated control. All treatments of brood were in re-capped cells with a non-treated control.
(DOC)

S2 Table Analyses of covariance (ANCOVA) of relative expression units (REU) on gene expression in European bees between different treatments. Buffer and homogenate injection treatments of adult bees were performed in hoarding cages with a non-treated control. *Varroa* parasitism of adult bees was conducted in queen cages with a non-treated control. All treatments of brood were in re-capped cells with a non-treated control.
(DOC)
