# Supplementary figures and images for: Effect of Varroa destructor, Wounding and Varroa Homogenate on Gene Expression in Brood and Adult Honey Bees
Source: PLoS One. 2017 Jan 12;12(1):e0169669. doi: 10.1371/journal.pone.0169669 (PMC5232351; doi:10.1371/journal.pone.0169669)

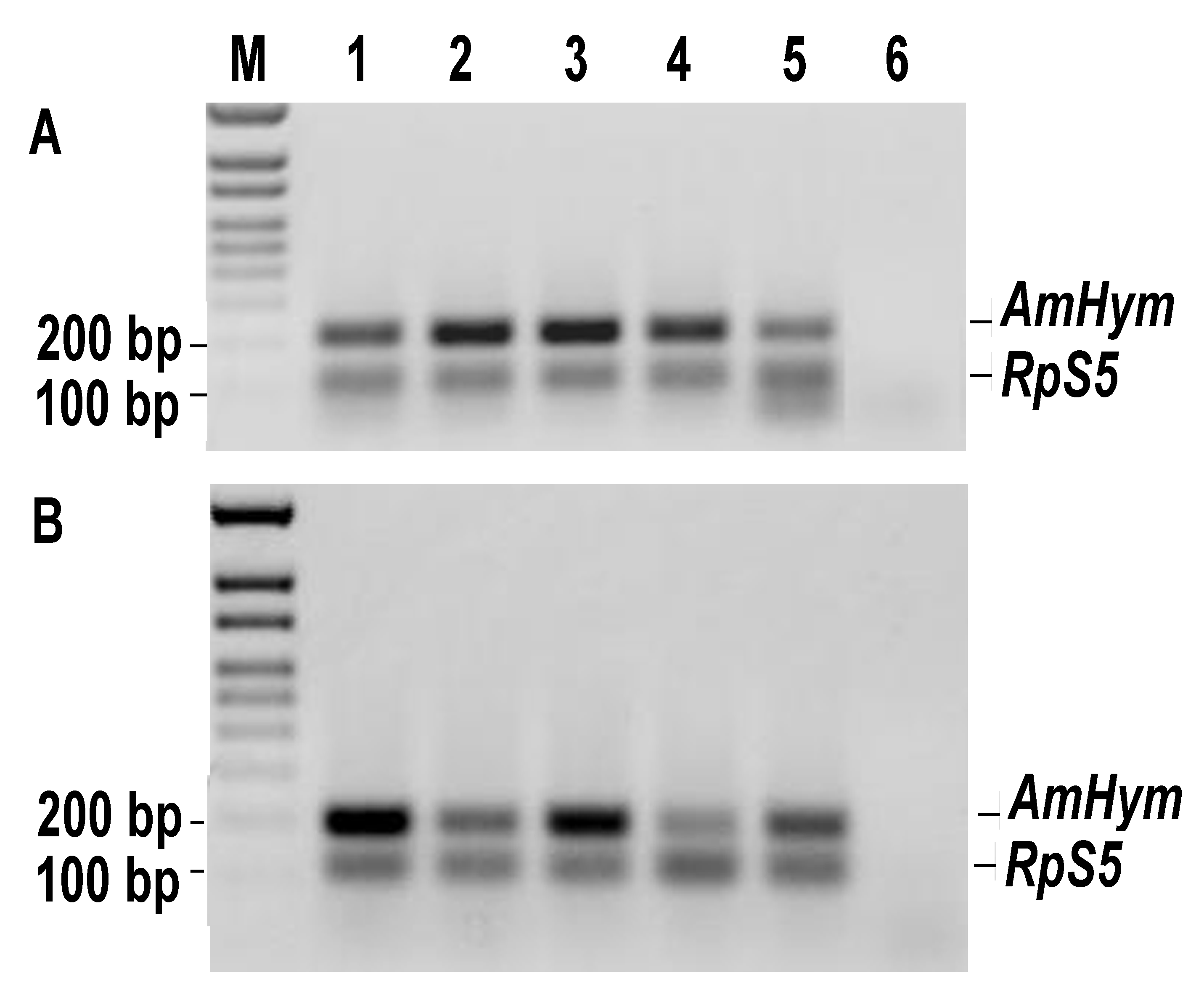

Supplement: S1 Fig — Gel picture of the co-amplification of AmHym and the housekeeping gene, RpS5, used to estimate relative expression in brood (A) and adult bees (B) in response to buffer injection at different hours post treatment (hpt). Lanes 1–5 show the control treatment at 0, 2, 12, 24 and 48 hpt, respectively. Lane 6 shows negative control with no DNA. Lane M (far left) is a 100 bp DNA ladder for both panels. (TIF) [file pone.0169669.s001.tif]

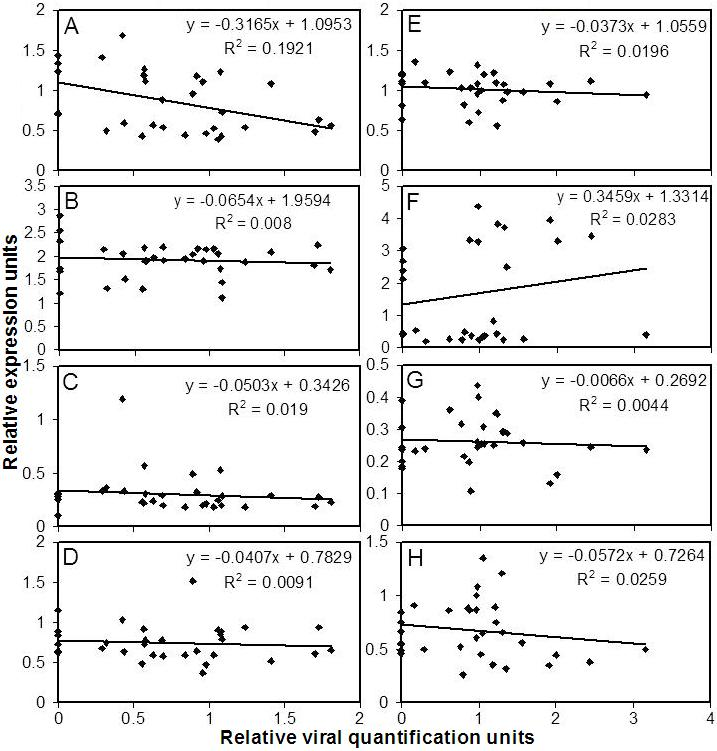

Supplement: S2 Fig — The panels are relative expression units of AmDef-1, AmHym, AmPuf68, AmVit2 in adult bees (A, B, C, D) and in brood (E, F, G, H), respectively. Thirty-two samples with different levels of viral quantification were used to generate the linear regression line and equation. (TIF) [file pone.0169669.s002.TIF]

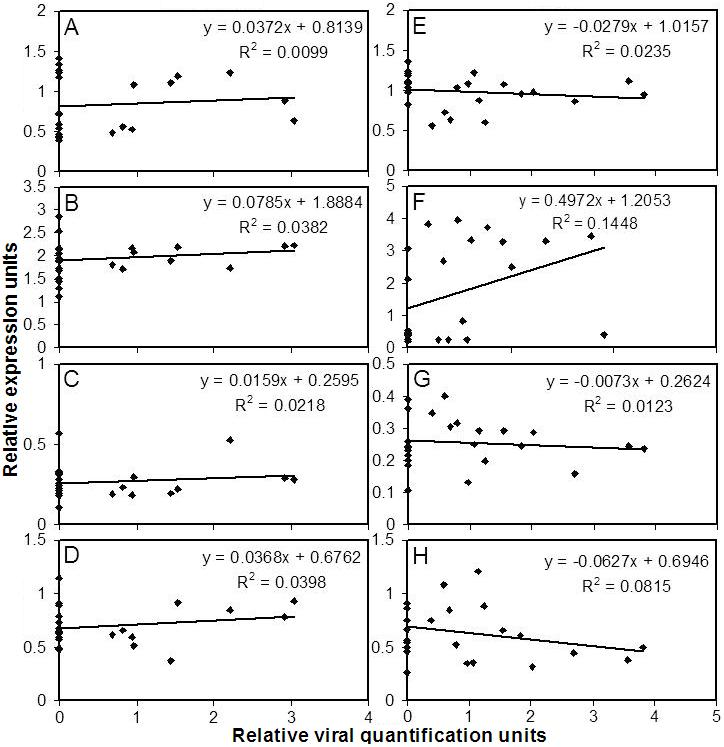

Supplement: S3 Fig — The panels are relative expression units of AmDef-1, AmHym, AmPuf68, AmVit2 in adult bees (A, B, C, D) and in brood (E, F, G, H), respectively. Twenty-four samples with different levels of viral quantification were used to generate the linear regression line and equation. (TIF) [file pone.0169669.s003.TIF]

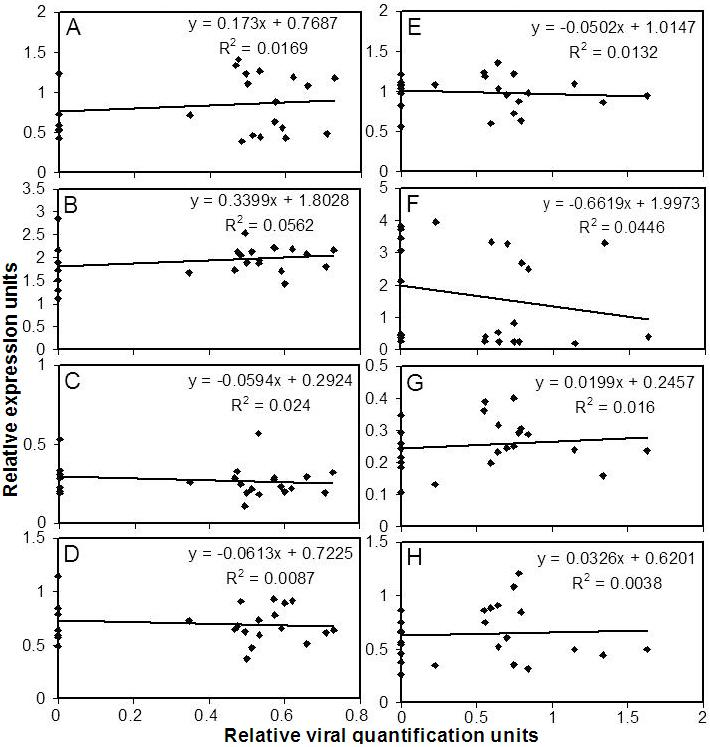

Supplement: S4 Fig — The panels are relative expression units of AmDef-1, AmHym, AmPuf68, AmVit2 in adult bees (A, B, C, D) and in brood (E, F, G, H), respectively. Twenty-four samples with different levels of viral quantification were used to generate the linear regression line and equation. (TIF) [file pone.0169669.s004.TIF]

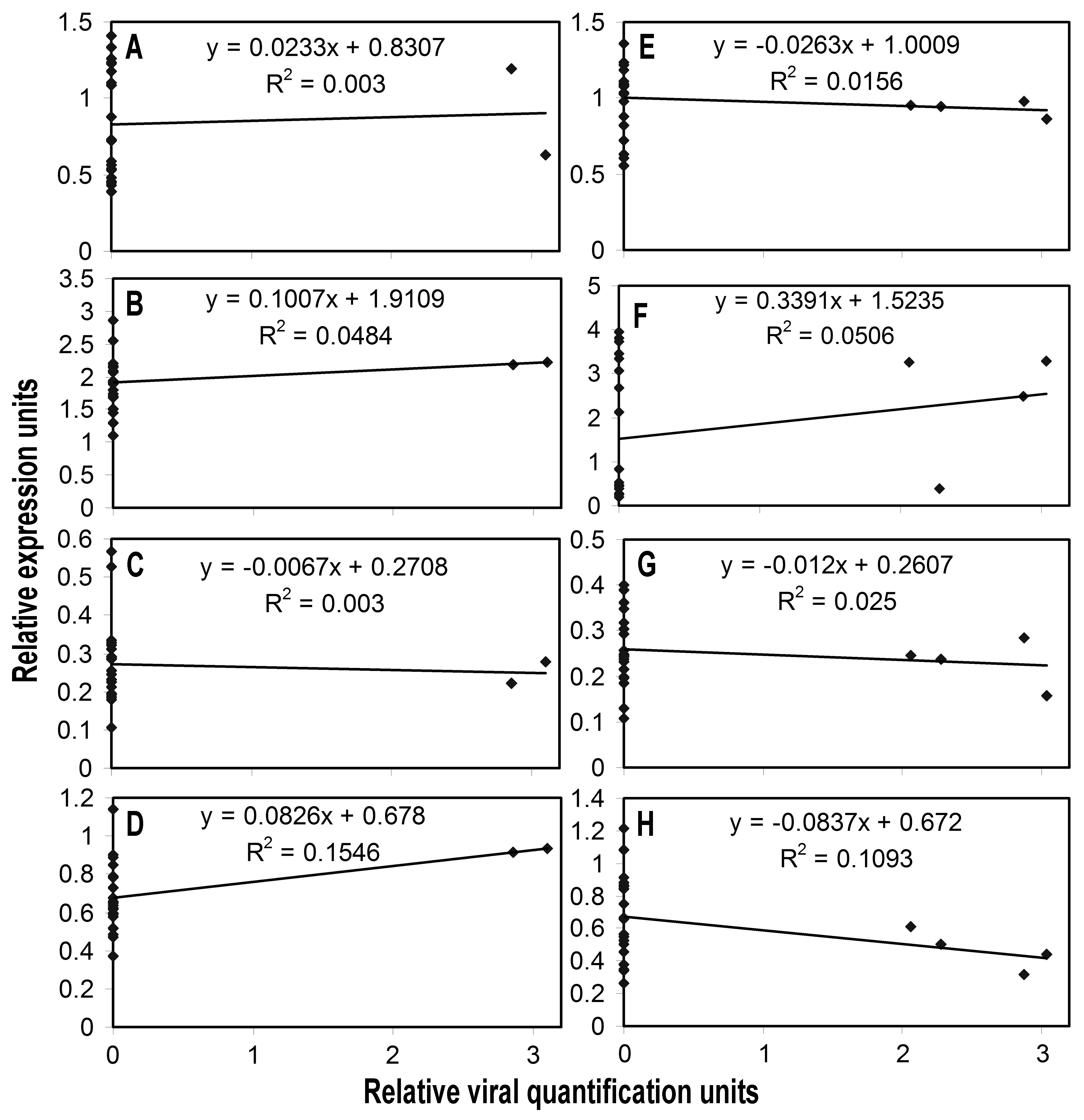

Supplement: S5 Fig — The panels are relative expression units of AmDef-1, AmHym, AmPuf68, AmVit2 in adult bees (A, B, C, D) and in brood (E, F, G, H), respectively. Twenty-four samples with different levels of viral quantification were used to generate the linear regression line and equation. (TIF) [file pone.0169669.s005.tif]
