# Supplementary material for: Effect of Varroa destructor, Wounding and Varroa Homogenate on Gene Expression in Brood and Adult Honey Bees
Source: PLoS One. 2017 Jan 12;12(1):e0169669. doi: 10.1371/journal.pone.0169669 (PMC5232351; doi:10.1371/journal.pone.0169669)
Supplement: S1 Table — Buffer and homogenate injection treatments of adult bees were performed in hoarding cages with a non-treated control. Varroa parasitism of adult bees was studied in queen cages with a non-treated control. All treatments of brood were in re-capped cells with a non-treated control. (DOC) [file pone.0169669.s006.doc]

**S1 Table. Analysis of variance (ANOVA) of relative expression units (REU) on gene expression in European bees at different time points. Buffer and homogenate injection treatments of adult bees were performed in hoarding cages with a non-treated control. *Varroa* parasitism of adult bees was studied in queen cages with a non-treated control. All treatments of brood were in re-capped cells with a non-treated control.**

| Gene | Time  (hpt) | Adult bees | | | | | Brood | | | |
| --- | --- | --- | --- | --- | --- | --- | --- | --- | --- | --- |
|  |  | Hoarding cage | | | Queen cage | |  |  |  |  |
|  |  | Con-  trol | Buf-  fer | Homo-  genate | Con-  trol | Var-  roa | Con-  trol | Buf-  fer | Homo-  genate | Var-  roa |
| *AmDef1* | 0  2  12  24  48 | NS | NS | c  a  ab  bc  c | NS | a  b  c  c  c | NS | NS | NS | abc  a  ab  c  bc |
| *AmHym* | 0  2  12  24  48 | NS | a  b  b  c  c | NS | NS | a  b  b  a  a | NS | d  bc  a  ab  cd | c  b  a  a  abc | a  ab  b  b  ab |
| *AmPuf68* | 0  2  12  24  48 | NS | a  b  b  b  b | a  b  b  b  b | ab  a  ab  b  ab | a  b  b  b  b | NS | NS | NS | NS |
| *AmVit2* | 0  2  12  24  48 | a  b  bc  cd  d | a  b  c  bc  bc | a  bc  bc  c  b | NS | a  b  bc  c  b | a  ab  ab  b  b | a  ab  ab  b  b | a  b  b  b  b | a  b  b  b  b |

Different letters indicate statistically significant differences of means between time points (hpt) (a>b>c>d) based on analyses of variance (Post Hoc Tests: LSD, Tamhane’s T2; α = 0.05) (IBM-SPSS v. 23; SPSS Inc., Chicago, IL, USA).

NS: non significant.

hpt: hours post treatment
