# Supplementary material for: Effect of Varroa destructor, Wounding and Varroa Homogenate on Gene Expression in Brood and Adult Honey Bees
Source: PLoS One. 2017 Jan 12;12(1):e0169669. doi: 10.1371/journal.pone.0169669 (PMC5232351; doi:10.1371/journal.pone.0169669)
Supplement: S2 Table — Buffer and homogenate injection treatments of adult bees were performed in hoarding cages with a non-treated control. Varroa parasitism of adult bees was conducted in queen cages with a non-treated control. All treatments of brood were in re-capped cells with a non-treated control. (DOC) [file pone.0169669.s007.doc]

**S2 Table.** Analyses of covariance (ANCOVA) of relative expression units (REU) on gene expression in European bees between different treatments. Buffer and homogenate injection treatments of adult bees were performed in hoarding cages with a non-treated control. *Varroa* parasitism of adult bees was conducted in queen cages with a non-treated control. All treatments of brood were in re-capped cells with a non-treated control.

| **Bees** | **Treatments** | **Genes (REU)** | | | |
| --- | --- | --- | --- | --- | --- |
|  |  | ***AmDef1*** | ***AmHym*** | ***AmPuf68*** | ***AmVit2*** |
| Adult bees | Control  Buffer  Homogenate  Control  Varroa | c  a  b  bc  d | a  c  b  b  c | a  c  cd  b  d | a  b  b  a  b |
| Brood | Control  Buffer  Homogenate  Varroa | ab  a  bc  c | c  b  a  c | b  a  ab  ab | a  ab  c  bc |

Different letters indicate statistically significant differences of means between different treatments based on analyses of covariance (ANCOVA) (a>b>c>d). Significant differences among means were separated with Fisher’s protected LSD (α = 0.05) (XLSTAT Version 2016.02.27390, Copyright Addinsoft 1995-2016, New York, NY 10001, USA).
